# Supplementary material for: Calcium dependence of neurotransmitter release at a high fidelity synapse
Source: eLife. 2021 Oct 6;10:e70408. doi: 10.7554/eLife.70408 (PMC8494478; doi:10.7554/eLife.70408)
Supplement: Transparent reporting form [file elife-70408-transrepform.pdf]

eLIFE
1st Floor
24 Hills Road
Cambridge CB2 1JP, UK
P 01223 855340
W elifesciences.org
T @elife

eLife's transparent reporting form

We encourage authors to provide detailed information *within their submission* to facilitate the interpretation and replication of experiments. Authors can upload supporting documentation to indicate the use of appropriate reporting guidelines for health-related research (see EQUATOR Network), life science research (see the BioSharing Information Resource), or the ARRIVE guidelines for reporting work involving animal research. Where applicable, authors should refer to any relevant reporting standards documents in this form.

If you have any questions, please consult our Journal Policies and/or contact us: editorial@elifesciences.org.

Sample-size estimation

- You should state whether an appropriate sample size was computed when the study was being designed
- You should state the statistical method of sample size computation and any required assumptions
- If no explicit power analysis was used, you should describe how you decided what sample (replicate) size (number) to use

Please outline where this information can be found within the submission (e.g., sections or figure legends), or explain why this information doesn't apply to your submission:

The impact of intracellular calcium concentration on pre-fusion steps (Fig. 1) is controversial at most synapses. At cerebellar mossy fiber boutons, previous indirect evidence argued for no calcium-dependence at all. Therefore, the effect size of the various parameters was not predictable prior to performing the experiments, and we could not do an explicit a priori power analysis. Because of the technical difficulties of these paired recordings, we performed eight experiments for each calcium concentrations. The reliability of the findings is further strengthened by uncaging experiments (Fig. 7).

Since the intracellular calcium dependence of neurotransmitter release was not studied before at this synapse, the shape of the dose-response curve and the scatter in these kind of data were not known, therefore the sample size was not computed when designing this study. Initially, capacitance measurements (n = 65) were performed until the shape of the curve appeared sufficiently defined (Fig. 2). Then, we performed high-frequency capacitance recordings (n = 15, Fig. 2) and paired recordings (n = 59, Fig. 3) to study the response at the very high and very low intracellular calcium, respectively. Taken into account the technical difficulty of these experiments and the observed data scatter, we performed these extra experiments until the dose-response curve was well defined. Therefore, the effect size of the various parameters was not predictable prior to performing the experiments, and we could not do an explicit a priori power analysis.

Replicates

- You should report how often each experiment was performed
- You should include a definition of biological versus technical replication

- The data obtained should be provided and sufficient information should be provided to indicate the number of independent biological and/or technical replicates
- If you encountered any outliers, you should describe how these were handled
- Criteria for exclusion/inclusion of data should be clearly stated
- High-throughput sequence data should be uploaded before submission, with a private link for reviewers provided (these are available from both GEO and ArrayExpress)

Please outline where this information can be found within the submission (e.g., sections or figure legends), or explain why this information doesn't apply to your submission:

For the data in Fig. 1, we performed 8 recordings obtained from 8 paired cells for each calcium concentrations. For obtaining the dose-response curves, 80 presynaptic recordings (obtained from 80 cells) and 59 simultaneous pre- and postsynaptic recordings (obtained from 43 paired cells) were performed. From the 43 paired cells, we included 16 consecutive recordings in which the preceding applied UV doses were very low, assuming minimal UV-induced cell toxicity and biological independence.

Exclusion criteria: insufficient signal-to-noise for fitting the initial phase of the recording.

This information can be found in the methods section (line 140-143) and the legends to Figs. 1 – 3.

Statistical reporting

- Statistical analysis methods should be described and justified
- Raw data should be presented in figures whenever informative to do so (typically when N per group is less than 10)
- For each experiment, you should identify the statistical tests used, exact values of N, definitions of center, methods of multiple test correction, and dispersion and precision measures (e.g., mean, median, SD, SEM, confidence intervals; and, for the major substantive results, a measure of effect size (e.g., Pearson's r, Cohen's d)
- Report exact p-values wherever possible alongside the summary statistics and 95% confidence intervals. These should be reported for all key questions and not only when the p-value is less than 0.05.

Please outline where this information can be found within the submission (e.g., sections or figure legends), or explain why this information doesn't apply to your submission:

Statistical analysis methods are reported in "Statistical analysis" section (line 512). The exact P-values are indicated in the figures above the boxplots. The statistical test and the sample size are reported in the corresponding figure legend.

(For large datasets, or papers with a very large number of statistical tests, you may upload a single table file with tests, Ns, etc., with reference to sections in the manuscript.)

Group allocation

- Indicate how samples were allocated into experimental groups (in the case of clinical studies, please specify allocation to treatment method); if randomization was used, please also state if restricted randomization was applied
- Indicate if masking was used during group allocation, data collection and/or data analysis

Please outline where this information can be found within the submission (e.g., sections or figure legends), or explain why this information doesn't apply to your submission:

Brain slices from different animals were randomly assigned to the experiment and no specific method of randomization was used. Masking could not be done during data collection. All collected data were quantifiable and blinding would not change any bias in the analyzed data.

Additional data files ("source data")

- We encourage you to upload relevant additional data files, such as numerical data that are represented as a graph in a figure, or as a summary table
- Where provided, these should be in the most useful format, and they can be uploaded as "Source data" files linked to a main figure or table
- Include model definition files including the full list of parameters used
- Include code used for data analysis (e.g., R, MatLab)
- Avoid stating that data files are "available upon request"

Please indicate the figures or tables for which source data files have been provided:

The code generated during this study will be available at GitHub
